# Probiotics and Fever Duration in Children With Upper Respiratory Tract Infections: A Randomized Clinical Trial

**DOI:** 10.1001/jamanetworkopen.2025.0669

**Published:** 2025-03-14

**Authors:** Silvia Bettocchi, Anna Comotti, Marina Elli, Valentina De Cosmi, Cristiana Berti, Ilaria Alberti, Alessandra Mazzocchi, Chiara Rosazza, Carlo Agostoni, Gregorio Paolo Milani

**Affiliations:** 1 Pediatric Unit, Foundation Istituto di Ricovero e Cura a Carattere Scientifico (IRCCS) Ca’ Granda Ospedale Maggiore Policlinico, Milan, Italy; 2 Occupational Health Unit, Foundation IRCCS Ca’ Granda Ospedale Maggiore Policlinico, Milan, Italy; 3 Company AAT–Advanced Analytical Technologies Srl, Fiorenzuola d’Arda, Italy; 4 Department of Food Safety, Nutrition and Veterinary Public Health, Istituto Superiore di Sanità, Rome, Italy; 5 Department of Clinical Sciences and Community Health, University of Milan, Milan, Italy

## Abstract

**Importance:**

Upper respiratory tract infections (URTIs) are prevalent in children, prompting frequent health care consultations, especially among those with fever. Probiotics show potential as an adjuvant treatment for URTIs, but evidence in children is limited.

**Objective:**

To evaluate the efficacy of a probiotic mixture containing *Bifidobacterium breve* M-16V, *Bifidobacterium lactis* HN019, and *Lactobacillus rhamnosus* HN001 in shortening fever duration among children with URTIs.

**Design, Setting, and Participants:**

This randomized clinical trial was conducted between November 19, 2021, and June 20, 2023, at the pediatric emergency department of the Ca’ Granda Ospedale Maggiore Policlinico in Milan, Italy. Patients between 28 days and 4 years of age with a fever (≥38.5 °C) and URTI were eligible. Exclusion criteria included recent probiotic use, chronic autoimmune diseases, immunosuppressive treatment, and requirement for hospitalization. Randomization was computer generated and assigned participants to either the intervention (probiotics) or control (placebo) group. Participants, parents or caregivers, and investigators were masked to the group assignments. The primary analysis followed the intention-to-treat approach.

**Interventions:**

The probiotic group received daily single dose of 0.5 mL probiotic mixture containing *Bifidobacterium breve* M-16V, *Bifidobacterium lactis* HN019, and *Lactobacillus rhamnosus* HN001 for 14 days. The placebo group received daily single dose of 0.5 mL placebo for 14 days.

**Main Outcomes and Measures:**

The primary outcome was fever duration, defined as the number of days between the first and the last days with fever.

**Results:**

Of the 128 patients enrolled (69 males [54%]; mean [SD] age 2.5 [1.3] years), 65 (51%) were randomly assigned to receive placebo and 63 (49%) to receive probiotics. The median (IQR) fever duration was shorter in the probiotic group than the placebo group (median [IQR], 3 [2-4] days vs 5 [4-6] days; adjusted risk ratio, 0.64; 95% CI, 0.51-0.80). Few mild adverse events were reported and did not significantly differ between the probiotic and placebo groups, including constipation (6 [16%] and 6 [12%]; *P* = .80) and abdominal pain (3 [8%] and 2 [4%]; *P* = .65).

**Conclusions and Relevance:**

In this randomized clinical trial, administering a probiotic mixture reduced fever duration by 2 days compared with placebo, with no meaningful safety concerns. The probiotic mixture under investigation could be an effective adjuvant for shortening fever duration in children with URTIs.

**Trial Registration:**

ClinicalTrials.gov Identifier: NCT06052540

## Key Points

**Question:**

Does a probiotic mixture containing *Bifidobacterium breve* M-16V, *Bifidobacterium lactis* HN019, and *Lactobacillus rhamnosus* HN001 reduce fever duration in children with upper respiratory tract infections (URTIs)?

**Findings:**

In this randomized clinical trial involving 128 children with URTIs, the probiotic mixture shortened the duration of a fever by 2 days, a statistically significant difference compared with placebo.

**Meaning:**

The findings suggest that probiotics are helpful in the treatment of pediatric URTIs.

## Teaser

This randomized clinical trial evaluates the efficacy and safety of a probiotic mixture containing *Bifidobacterium breve* M-16V, *Bifidobacterium lactis* HN019, and *Lactobacillus rhamnosus* HN001 in reducing fever duration among children as young as 28 days with upper respiratory tract infections.

## Introduction

Upper respiratory tract infections (URTIs) are highly reported in the pediatric population, representing approximately 90% of the total respiratory infections worldwide.^1,2^ It is common for a child to have 5 to 8 URTIs a year, especially in the first 5 years of life.^3^ These conditions are the predominant reason for seeking consultation with a general practitioner,^4^ with a hospital admission rate greater than 1%.^5^ URTIs are triggered by different infectious agents, primarily respiratory viruses.^6^ Symptoms of URTIs usually peak in 3 to 5 days; resolve within 14 days; and include nasal congestion and discharge, sore throat, eye redness or drainage, cough, hoarseness, irritability, decrease in appetite, sleep disturbance, and fever.^7^ Fever, in particular, is a common feature in patients affected by URTIs with a substantial impact on the child’s well-being as well as on physical and scholastic activities. Moreover, it is a potential source of concern for both physicians and caregivers and is associated with an increased, and sometimes inappropriate, use of antibiotics.^6,8^ Currently, there is no evidence-based routinary treatment for URTIs. The use of antipyretics transiently decreases the body temperature but has no effect on the overall duration of fever.^9^ Antibiotics are effective only in a limited number of URTIs.^10^

In past decades, the administration of probiotics has emerged as a new potential approach to managing infectious diseases. The most widespread application of these supplements has been the mitigation of gastrointestinal symptoms.^11^ However, the interplay between gut microbiome, inflammatory processes, and immune response suggests the possible role of probiotics in infectious conditions not limited to the gastrointestinal tract.^12,13^

Few data are available on the role of probiotics in children with respiratory tract infections. Hence, the primary aim of this trial was to evaluate the efficacy of a probiotic mixture containing *Bifidobacterium breve* M-16V, *Bifidobacterium lactis* HN019, and *Lactobacillus rhamnosus* HN001 in shortening fever duration among children with URTIs.

## Methods

### Trial Design

This investigator-initiated triple-blind, placebo-controlled randomized clinical trial was conducted between November 19, 2021, and June 20, 2023, at the pediatric emergency department (ED) of the Fondazione IRCCS Ca’ Granda Ospedale Maggiore Policlinico in Milan, Italy. The Milan Area 2 Institutional Ethics Committee approved the trial protocol (Supplement 1). The trial complied with the Declaration of Helsinki.^14^. Written informed consent was obtained from the parents or legal caregivers of child participants. We followed the Consolidated Standards of Reporting Trials (CONSORT) reporting guideline.

At the time the study began, we understood that trial registration was not required under Italian law for non-pharmacological interventions involving food supplements, which are classified as foods rather than drugs. However, we did register the trial in ClinicaTrials.gov, albeit after the trial was conducted. After discussion with colleagues during the conduction of the study, the issue of lack of public registration emerged and therefore we decided to register it as soon as it was noticed. This delay was not intended to influence the conduct or reporting of the study. An interim analysis was conducted when approximately 30% of the target sample size had completed the study. This analysis was performed to assess the possibility of early termination based on the available data as requested by our ethics committee.

### Participants

Eligible participants were at least 28 days to 4 years of age who presented with fever (rectal temperature ≥38.5 °C) and were diagnosed with URTIs by the pediatrician on duty at the ED at the time of enrollment. Patients with diarrhea, history of probiotic supplementation in the previous 2 weeks, chronic autoimmune diseases, ongoing treatment with immunosuppressive drugs, and hospitalization requirement were excluded.

Immediately after discharge from the ED, caregivers were approached by 1 of the researchers and invited to participate in the trial. Then, self-reported data on patient age, sex, race and ethnicity, and birth weight were collected. Body height (Harpenden stadiometer) and weight (Tanita TL-150 MA; Sensor Medics) were measured by a trained investigator. Race and ethnicity data were collected because evidence suggests that the human microbiome may vary according to these demographic factors. Inclusion of these variables enhances the generalizability of the findings.

### Randomization

After enrollment and baseline data collection, participants were randomly assigned using a computer-based number generator to 1 of 2 groups: intervention group (to receive daily single dose of 0.5 mL or 1.5 g probiotic mixture containing *Bifidobacterium breve* M-16V, *Bifidobacterium lactis* HN019, and *Lactobacillus rhamnosus* HN001 for 14 days) and control group (to receive daily single dose of 0.5 mL or 1.5 g placebo for 14 days). Throughout the duration of the trial, all investigators, participants, caregivers, and data analysts were blinded to group assignment.

Probiotic sticks contained maltodextrin, *Bifidobacterium breve* M-16V LMG 23729 (1 × 10^9^ CFUs [colony-forming units]), *Bifibidobacterium lactis* HN019 ATCC SD5674 (1 × 10^9^ CFUs), and *Lactobacillus rhamnosus* HN001 ATCC SD5675 (1 × 10^9^ CFUs). Probiotic oily drops contained medium-chain triglycerides, *Bifidobacterium breve* M-16V LMG 23729 (1 × 10^9^ CFUs), *Bifibidobacterium lactis* HN019 ATCC SD5674 (1 × 10^9^ CFUs), *Lactobacillus rhamnosus* HN001 ATCC SD5675 (1 × 10^9^ CFUs), vegetal fats (Karité butter), and an emulsifier agent (monoglycerides and diglycerides of fatty acids [E471]). Placebo sticks contained only maltodextrin, while placebo oily drops contained medium-chain triglycerides, vegetal oil, emulsifier E471 (monoglycerides and diglycerides of food fatty acids; vegetal sources), and maltodextrin-like strain or replacement.

Both probiotic formulations are commercially available in Italy and can be purchased by consumers without any prescription. Probiotic and placebo formulas were produced according to the EU criteria (Regulation [EU] No. 609/2013 and No. 1333/2008). The probiotic and placebo formulations were indistinguishable by appearance, color, or flavor and were provided as stick or drops. Caregivers were instructed to choose sticks or drops based on the patient’s age and preference to encourage compliance.

### Procedures

Caregivers were thoroughly instructed to measure rectal body temperature at least 3 times a day (once in the morning, once in the afternoon, and once in the evening or night) until fever disappearance. Fever disappearance was defined as a rectal temperature less than 38.5 °C for at least 24 hours without the use of antipyretics. Follow-up assessments included a telephone call by one of the investigators (S.B.) approximately 7 days after enrollment to verify or inquire about supplementation compliance, fever duration, prescription of antibiotics in visits after discharge, and adverse effects. In cases of persistent fever, follow-up telephone calls were conducted every 7 days.

### Outcomes

The primary outcome was fever duration, defined as the number of days between the first and the last days with fever, as reported by caregivers. Additionally, we analyzed fever duration from the day of enrollment, calculated as the number of days between the last reported fever day and the day of enrollment.

The secondary outcomes were the incidence of diarrhea in patients prescribed antibiotics and the number of patients prescribed antibiotics during follow-up visits after ED discharge. Diarrhea was defined as the passage of 3 or more loose or watery stools within a 24-hour period.

Safety and tolerability were assessed based on reported adverse effects. Constipation was defined as 2 or fewer defecations per week, fecal incontinence after bowel control acquisition, pain or difficulty during defecation, or presence of large-diameter stools that may obstruct the toilet. Abdominal pain referred by patients to caregivers was also recorded.

### Statistical Analysis

The mean (SD) fever duration in children with URTIs in emergency settings was estimated to be 4 (2.6) days.^15^ A reduction greater than 36% (ie, at least >1.5 SD of 95% of the observations) was considered to be clinically significant. To detect this reduction with a type I error of 0.05 and a type II error of 0.20, a sample size of 48 participants per group was calculated. To account for a potential dropout rate of approximately 33%, we recruited a total of 128 patients.

Continuous variables were summarized as means (SD) or medians (IQR), while categorical variables were presented as frequencies and percentages. Normality of continuous variables was evaluated using the Shapiro-Wilk test. Group differences were analyzed using unpaired, 2-tailed *t* tests for normally distributed continuous variables, Wilcoxon rank sum tests for nonnormally distributed continuous variables, and χ^2^ or Fisher exact tests for categorical variables. We compared the baseline characteristics between the dropout group and the treament-adherent group (those who adhered either fully or partially).

The primary analysis followed the intention-to-treat approach, including participants who fully adhered to the intervention protocol and participants who partially followed the protocol (ie, assumed the supplementation for less than 14 days and/or started to take it after more than 24 hours after enrollment). To explore potential differences, a per-protocol analysis, including only participants who fully adhered to the treatment protocol, was also performed.

The median difference of fever duration between placebo and probiotic groups was tested using the Wilcoxon test. A Poisson regression model was used to investigate the association between the type of supplementation (independent variable) and fever duration (dependent count variable), adjusting for age, sex, and antibiotic intake as potential confounders. From this model, the rate ratio (RR) and corresponding 95% CI were calculated; the RR measured the association between supplementation type and fever duration. To assess the assumptions of the Poisson model, we verified that the mean and variance were comparable.

Adverse effects were compared between the placebo and probiotic groups, with frequencies and percentages calculated for each group. Since there were no missing data, no imputation technique was applied.

A 2-sided *P* < .05 was considered statistically significant. Analyses were performed from September 2023 to February 2024 using R, version 4.3.0 (R Project for Statistical Computing).

## Results

Characteristics of the 128 enrolled patients are summarized in Table 1. Participants included 69 males (54%) and 59 females (46%), with a mean (SD) age of 2.5 (1.3) years. The race and ethnicity were 5 Asian (4%), 2 Eurasian (1%), 13 Hispanic (10%), 8 North African (6%), and 100 White (79%) children. Thirty-three patients (26%) were receiving antibiotics at enrollment. Participants were allocated to either the probiotic group (63 [49%]) or the placebo group (65 [51%]). The Figure shows the trial flow diagram.

**Table 1.** Baseline Characteristics of the Enrolled Population^a^

Characteristic	Group, No. (%)	
	Placebo (n = 65)	Probiotic (n = 63)
Age, mean (SD), y	2.5 (1.3)	2.6 (1.3)
Sex		
Male	36 (55)	33 (52)
Female	29 (45)	30 (48)
Race and ethnicity^b^		
Asian	1 (2)	4 (6)
Eurasian	1 (2)	1 (2)
Hispanic	7 (11)	6 (10)
North African	4 (6)	4 (6)
White	52 (80)	48 (76)
Nutritional status		
Normal weight	60 (92)	58 (92)
Underweight	3 (5)	1 (2)
Overweight	2 (3)	4 (6)
Fever before enrollment, median (IQR), d	2 (1-3)	2 (1-3)
Antibiotic at enrollment	16 (25)	17 (27)

^a^ The World Health Organization (WHO) reference charts were used to calculate *z* scores and percentiles for weight for age, weight for length, and body mass index (calculated as weight in kilograms divided by height in meters squared). WHO criteria were consulted to classify child nutritional status.

^b^ Race and ethnicity were self-identified by parents or caregivers.

**Figure.**
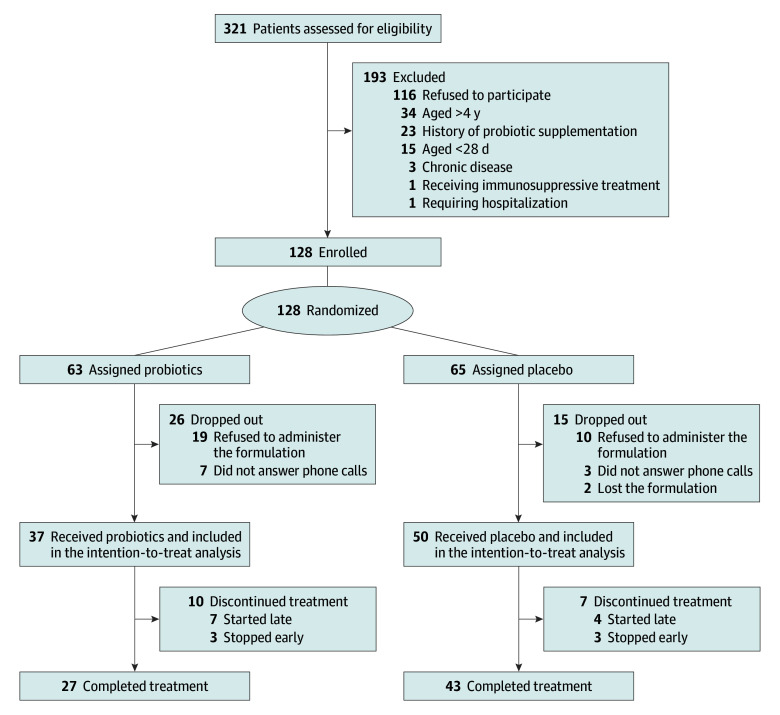
Participant Flow Diagram

Seventy patients (55%) were fully adherent with the treatment protocol, 17 (13%) were partially adherent, and 41 (32%) dropped out of the trial. eTable 1 in Supplement 2 summarizes characteristics of the total, intention-to-treat, and per-protocol sample. We found no significant differences by sex, age, and race and ethnicity between those (partially or fully) adherent to the treatment protocol and those who dropped out (eTable 2 in Supplement 2). The median (IQR) fever duration in the intention-to-treat sample was 4 (3-5) days. Table 2 shows the distribution of the total fever duration in the 2 treatment groups. Participants receiving probiotics presented a significantly lower fever duration compared with those receiving placebo (median [IQR], 3 [2-4] days vs 5 [4-6] days; *P* < .001). The mean (4.5) and SD (5.3) of the dependent variable were approximately equal, indicating no significant overdispersion and supporting the appropriateness of the Poisson model. The adjusted Poisson regression model indicated that the total fever duration was shorter in the probiotic group compared with the placebo group (RR, 0.64; 95% CI, 0.51-0.80). Similar findings were observed in the per-protocol analyses and the analysis limited to fever duration after enrollment (eTables 3-5 in Supplement 2).

**Table 2.** Total Fever Duration (Intention-to-Treat Analysis)

Group	No. of patients (%)	Fever duration, median (IQR), d	*P* value^a^
Total sample	87 (100)	4 (3-5)	NA
Placebo group	50 (57)	5 (4-6)	<.001
Probiotic group	37 (43)	3 (2-4)	NA

Abbreviation: NA, not applicable.

^a^ Probiotic vs placebo groups. *P* value was calculated using Wilcoxon rank-sum tests.

Among patients receiving antibiotics at enrollment, none in the intervention group and 3 in the control group developed diarrhea. Among patients discharged without any antibiotics, 1 of 46 (2%) in the probiotics group and 6 of 49 (12%) in the placebo group were prescribed antibiotics during follow-up visits after ED discharge (*P* = .16).

Reported adverse effects were diarrhea (4 [5%]), constipation (12 [14%]), and abdominal pain (5 [6%]). The distribution did not significantly differ between the probiotic and placebo groups, including constipation (6 [16%] and 6 [12%]; *P* = .80) and abdominal pain (3 [8%] and 2 [4%]; *P* = .65) (Table 3).

**Table 3.** Adverse Events in the Placebo and Probiotic Groups

Event	Events, No. (%)			*P* value^a^
	Total No. (%)	Placebo group (n = 50)	Probiotic group (n = 37)	
Diarrhea	4 (5)	4 (8)	0	.13
Constipation	12 (14)	6 (12)	6 (16)	.80
Abdominal pain	5 (6)	2 (4)	3 (8)	.65

^a^ *P* values were calculated using χ^2^ or Fisher exact test as appropriate.

## Discussion

This trial showed for the first time, to our knowledge, the efficacy of a probiotic mixture containing *Bifidobacterium breve* M-16V, *Bifidobacterium lactis* HN019, and *Lactobacillus rhamnosus* HN001 in shortening fever duration by approximately 2 days in children with URTIs. The administration of these supplements did not reduce the incidence of diarrhea in children receiving antibiotics or the prescription of antibiotics after ED discharge. Overall, the probiotic mixture was safe, and few mild adverse events were observed.

The use of probiotics for respiratory diseases has been receiving increased attention, although most available data are limited to the prevention of respiratory infections. A systematic review published in 2020 reported that probiotics might be associated with reduced occurrence of URTIs and symptom severity in children.^16^ However, recent trials have yielded conflicting evidence. Investigations conducted in Italy and Slovakia have highlighted divergent outcomes regarding the effect of probiotics on URTI incidence.^1,17^ Similar inconsistencies have been observed in studies from Finland and Scotland.^18,19^

Given the inconclusive data on prevention, finding effective treatments might be crucial to alleviate the burden of these infections on physical and scholastic activities, school absenteeism, and hospitalizations. The present trial showed that patients treated with a mixture of *Bifidobacteria* and *Lactobacilli* presented an overall reduction of fever duration by approximately 2 days compared with patients treated with placebo. These data appear in contrast with results of a recent systematic review of 3 trials on probiotics as treatment for URTIs in childhood that did not support the routinary administration of these supplements.^20^ Among the studies in the systematic review, 1 trial on children with pharyngitis found that *Limosilactobacillus reuteri* reduced fever by approximately 2 days. However, that trial enrolled children both with and without fever and did not perform subanalyses limited to feverish children.^21^ The second trial, involving infants hospitalized for respiratory tract infections (bronchiolitis, pneumonia, croup, tonsillopharyngitis, and laryngitis), found that *Bifidobacterium lactis* Probio-M8 was associated with a shorter duration of symptoms and hospital stay; no intention-to-treat analysis was performed.^22^ The third trial, which provided a multistrain mixture, did not observe any effect on respiratory symptoms of children affected with bacterial pneumonia.^23^ No adjustment for potential confounders was made in these 3 trials. In contrast to the previously discussed studies, the present trial included children exclusively affected by a URTI with fever, and the effects of the probiotic mixture were consistent across per-protocol and intention-to-treat analyses. Furthermore, the fever reduction remained consistent even after adjusting for age, sex, and antibiotic use.

The findings align with growing evidence on the immunomodulatory effects of probiotics. Several mechanisms have been proposed to explain the interaction between probiotics, the immune system, and pathogens. Several studies have shown that a probiotic mixture can enhance humoral immunity, especially the activity of macrophages and dendritic cells, and increase the production of antibodies (such as immunoglobulin [Ig] A, IgG, and IgM).^10,24^ Furthermore, probiotics have shown antiviral activity against prevalent respiratory viruses, such as influenza, rhinovirus, respiratory syncytial virus, and coronavirus.^25^ Previous studies have pointed out that species of probiotics used in this trial, such as *Bifidobacteria* and *Lactobacilli*, can modulate the expressions of interleukin 1, interleukin 6, and tumor necrosis factor,^26,27,28^ which play a key role in the development of fever.^29^ These mechanisms may partly explain the significant reduction in fever duration observed in this trial.

A recent trial pointed out the role of probiotics in preventing antibiotic-associated diarrhea.^30^ There was no significant difference in incidence of diarrhea between the 2 groups. A similar finding was observed for antibiotic prescription after discharge. However, these data need to be confirmed in larger studies.

The mixture of probiotics was well-tolerated and without any relevant adverse effects. These findings support the use of probiotics in children without any underlying risk for severe adverse effects (eg, those with immunosuppressed conditions) and are consistent with findings in previous literature.^31^

### Limitations

This trial has limitations. First, we did not investigate specific infectious diseases (eg, influenza) or differentiate between bacterial and viral infections. Moreover, the diagnosis of URTI and antibiotic prescription were not standardized but relied on medical evaluation of on-duty physicians. However, the inclusion of all children with URTIs in a clinical setting enhances the generalizability of the results. In addition, the trial was conducted in a real-life setting where standardizing antipyretic administration (type, dose, schedule) was not feasible and would have reduced external validity. Second, the participants did not receive supplementation at the same stage of the disease course given the setting of the trial. To account for this issue, we also considered fever duration from the day of the trial initiation, adjusting for the number of days with fever before supplementation. Third, caregivers measured temperature at home, without supervision from the research staff. To minimize potential errors, caregivers received precise instructions from the staff, including consistent instrument use and temperature measurements at a specific time of the day. Protocol adherence was also monitored during the telephone follow-up. Automated temperature monitoring or wearable thermometers might be used in future trials. Fourth, the trial was conducted in only 1 ED. Fifth, the sample size calculation was based on the primary outcome, and effects from other outcomes might not have been detected due to the limited number of enrolled patients. In addition, we acknowledge the high dropout rate in our trial, especially in the probiotic group (58%). Several participants declined to administer the supplement or discontinued early, which we attribute to the real-life, non-hospital-based setting of the administration. Despite this limitation, we adopted an intention-to-treat approach, and also conducted per-protocol analyses, which showed consistent results. While a high dropout rate may affect statistical power and generalizability, we believe that our findings remain robust.

## Conclusions

This randomized clinical trial indicates that administering a probiotic mixture containing *Bifidobacterium breve* M-16V, *Bifidobacterium lactis* HN019, and *Lactobacillus rhamnosus* HN001 resulted in shorter fever duration by 2 days compared with placebo in children with URTIs. No safety and tolerability issues were observed.

## Notes

Supplement 1.
Trial Protocol

Supplement 2. **eTable 1.** Baseline Characteristics of Children Included in the Total Sample, Intention-to-Treat (ITT) Analysis and Per-Protocol (PP) Analysis **eTable 2.** Demographic Characteristics of Dropout and Compliant Subjects **eTable 3.** Per Protocol Analysis (Median Difference) **eTable 4.** Differences Between Fever Duration Between Placebo and Probiotic Groups Considering as Outcome the Number of Days With Fever After Enrollment **eTable 5.** Poisson Regression Model of Probiotics on Duration of Fever After Enrollment

Supplement 3.
Data Sharing Statement
