# Supplementary material for: Probiotics and Fever Duration in Children With Upper Respiratory Tract Infections: A Randomized Clinical Trial
Source: JAMA Netw Open. 2025 Mar 14;8(3):e250669. doi: 10.1001/jamanetworkopen.2025.0669 (PMC11909606; doi:10.1001/jamanetworkopen.2025.0669)
Supplement: Supplement 1. — Trial Protocol [file jamanetwopen-e250669-s001.pdf]

Dipartimento Area Materno Infantile
S.C. Pronto Soccorso Pediatrico - Direttore: Prof. Carlo V. Agostoni
Tel. 02 5503.2349
E-mail: prontosoccorsopediatrico@policlinico.mi.it

The original version of the full study protocol was in Italian (attached below). We provide a translated version in English.

Protocol

Effect of Supplementation with a Mixture of Probiotic Strains (Bifidobacterium breve M-16V, Bifidobacterium lactis HN019, Lactobacillus rhamnosus HN001) in Febrile Children with Upper Respiratory Tract Infections

Acronym: PROBIOS2021

Sponsor: Fondazione IRCCS Ca' Granda Ospedale Maggiore Policlinico
Via Sforza 28, 20122 Milano, Italy

Coordinating Center: Pediatric Emergency Room
Fondazione IRCCS Ca' Granda Ospedale Maggiore Policlinico
Via della Commenda 9, 20122 Milano, Italy

Principal Investigator: Carlo Agostoni

Document Type: Non-Pharmacological Interventional Study Protocol

Version Number: v.1.0

Date: 12/05/2021

CONFIDENTIALITY STATEMENT
All information presented in this document will be considered confidential and will remain the exclusive property of Fondazione IRCCS Ca' Granda Ospedale Maggiore Policlinico. The use of such confidential information should be limited to the recipient for the agreed purpose and must not be disclosed, published, or otherwise communicated to unauthorized persons for any reason in any form without prior written consent from Fondazione IRCCS Ca' Granda Ospedale Maggiore Policlinico.

FLOWCHART

	**Assignment/ Randomization**		
**Period**	**Visit 1 (t0)**	**End of Intervention (t1)**	**Follow-up (t2)**
Enrollment			
Informed consent	x		x
Inclusion/exclusion criteria	x		
CRF completion	x	x	
Intervention administration			
Probiotic mixture group	x		
Placebo group	x		
Measurements			
Anamnestic and anthropometric variables	x	x	
Fever measurement	x	x	
Stool collection	x	x	x
Gut microbiota analysis	x	x	x

INDEX

1. INTRODUCTION

    1.1 Background and Rationale

2. TRIAL OBJECTIVE

    2.1 Primary Objective

    2.2 Secondary Objective(s)

3. STUDY DESIGN

    3.1 Study Design

    3.2 Inclusion Criteria

3.3 Exclusion Criteria

4. STUDY PROCEDURES

4.1 Intervention

4.2 Randomization

4.3 Blinding

5. ENDPOINTS

5.1 Primary Endpoint

5.2 Secondary Endpoint

6. STUDY DURATION/TIMELINE

7. STATISTICAL ANALYSIS

7.1 Sample Size

7.2 Data Analysis

8. ADVERSE EVENTS
9. RISK/BENEFIT ASSESSMENT
10. STUDY MANAGEMENT

10.1 Data Collection and Management

10.2 Regulatory and Ethical Considerations

10.2.1 Competent Authority Approval

10.2.2 Ethics Committee Approval

10.2.3 Informed Consent

10.3 Investigator Duties

10.4 Study Monitoring

10.5 Quality Assurance

10.6 Study Closure

10.7 Document Archiving

10.8 Information Disclosure

10.8.1 Confidentiality

10.8.2 Publications

10.9 Intellectual Property Rights

11. Compensation for Damages
12. Protocol Amendments
13. Financial Agreements
14. Conflict of Interest Disclosure
15. References
16. Appendix

Responsibilities (Role of the Sponsor and Collaborators)

- **Carlo Agostoni:** Principal Investigator

External Collaborations (Biological Sample Analysis, Data Analysis, Diagnostic Procedures, etc.)

Institution	Operational Unit	Participant Name	Role and Functions in the Study
AAT – Advanced Analytical Technologies Srl	Research and Development Lab	Dr. Lorenzo Morelli	Characterization of the microbiota from fecal analysis
		Dr. Marina Elli	Generation of the random allocation sequence and final decoding

Timelines

Study Status	Planning
Study Start	01/09/2021
Enrollment Closure	28/02/2023
Final Study Report	31/08/2023
12-Month Follow-Up Start	01/05/2023
Enrollment Closure	31/05/2024
Final Study Report	31/12/2024

1. INTRODUCTION

1.1 Background and Rationale

The human body is the habitat of billions of microorganisms that together form an ecosystem called the microbiota. The gastrointestinal tract is the compartment with the highest density of microbial cells and is the most studied portion to date (1). The gut microbiota is increasingly considered an "invisible organ" of the human body essential for host health; a symbiosis is created between bacteria and the host where the body provides nutrients necessary for bacterial survival and development, and microorganisms produce substances necessary for the development and well-being of the individual (2). At birth, the newborn's gastrointestinal tract is virtually sterile but is quickly exposed to colonization by opportunistic bacteria from the maternal vaginal, fecal, or skin microbiota; indeed, there is a significant similarity between the newborn's microflora and the mother's, consistent with previous evidence of vertical transmission of microorganisms (3). Thus, the mode of delivery influences the composition of the newborn's microbiota. During the first 24 hours of extrauterine life, the gastrointestinal tract is mainly colonized by facultative anaerobes such as Streptococci, Enterococci, and Escherichia coli; later, obligate anaerobes such as Bifidobacteria and Lactobacilli settle in (4). The development of the bacterial flora is also influenced by the infant's diet, i.e., breastfeeding or formula feeding. Numerous studies have demonstrated the different composition of the intestinal microbiota of breastfed versus formula-fed individuals. Breast milk is rich in monosaccharides such as lactose, glycolipids, glycoproteins, sialic acid, fucose, oligosaccharides, and N-acetylglucosamine (3). These components, called prebiotics, reach the intestine undigested and serve a defensive function by creating a shield

against pathogenic bacteria. Moreover, since bacteria can ferment milk compounds, there is an increase in fermentative microorganisms with a parallel decrease in putrefactive ones and a reduction in pH. Under these conditions, there is an improvement in digestive and absorption functions, a relative stimulation of the immune system, and a reduction in the risk of allergies and vitamin deficiencies (5).

The introduction of solid foods complementary to milk has been correlated with a reduction in variability of the gut microbiota among individuals (6). However, the maturation of the intestinal microbial community is generally reached around 2 years of age (7). The gut microbiota tends to maintain a certain homeostatic state throughout life; that of children, unlike that of adults, which is considered relatively stable, is highly dynamic and prone to alterations. Numerous factors can modulate this maturation, including the family environment in which the child is placed and other influences related to culture, traditions, and geographic area. Another external factor that can alter the composition of the microbiota is inflammation. Inflammation is a biological response to the activation of the immune system after contact with infectious external agents.

Probiotics, according to the WHO (World Health Organization), are live microorganisms which, when administered in adequate amounts, confer a health benefit on the host (8). It is known that probiotics can strengthen the immune response by restoring symbiosis within the gut microbiota, thereby aiding immune protection against various pathogens, including those responsible for respiratory infections (9).

Finally, exposure to antibiotics can also alter the composition of the gut microbiota. Antibiotics (substances produced by bacteria, fungi, or synthetic origin capable of inhibiting the growth of other microorganisms) are the most commonly used drugs in neonatal and pediatric populations in Western countries. Antibiotic therapies can alter the initial microbial composition and cause loss of colonization resistance (3). Consequently, the host may become susceptible to colonization by one or more pathogens. Since the composition of the infant gut microbiota is very important for maintaining an optimal state of health, it is equally important to accurately identify the bacterial species residing in the gastrointestinal tract at various times of life, both in good health and in

pathological conditions. The first years of life are thus the most critical and fundamentally important period for the stabilization of the microbiota (4).

2. TRIAL OBJECTIVE

Evaluation of the Effects of the Probiotic Formulation Ofmom Synteract Bimbi on Children's Health Status. The hypothesis posits that children receiving the probiotic mixture will experience a shorter duration and lower intensity of fever compared to those in the placebo group. Furthermore, the probiotic formulation may contribute to the prevention of gastrointestinal disturbances commonly associated with antibiotic therapy.

2.1 Primary Objective

The primary objective of the study is to assess the effects of the probiotic formulation *Ofmom Synteract Bimbi* on the intensity and duration of fever in response to upper respiratory tract infections.

2.2 Secondary Objective(s)

To evaluate the effect of supplementation with the probiotic mixture on:

- Analysis of the impact of probiotic products on the gut microbiota of treated subjects
- Analysis of different outcomes in patient subgroups
- Analysis of gut microbiota after 12 months from enrollment

3. STUDY DESIGN

3.1 Study Design This is a prospective, biological, randomized, controlled, non-pharmacological interventional study with a 1:1 allocation ratio in triple-blind conditions. The study duration for each patient will be 14 days (administration of *Ofmom Synteract Bimbi*, one stick per day or five drops per day). Probiotics will be administered after the first stool sample is collected. The study will be proposed to parents during their visit to the Pediatric Emergency Department, where they

will complete informed consent. After 12 months, the parents of the recruited patients will be recontacted and offered the third stool collection (T2). Parents will complete informed consent at this time as well.

3.2 Inclusion Criteria Patients who meet the following criteria will be included and asked to participate voluntarily:

- Access to the Pediatric Emergency Department with fever and upper respiratory tract infection.
- Age > 28 days and ≤ 4 years.
- Informed consent signed by parents or guardians.

3.3 Exclusion Criteria Patients will be excluded if they meet any of the following criteria:

- Presence of diseases associated with immunosuppression or ongoing immunosuppressive treatments.
- Hospitalization.
- Diarrhea at the time of enrollment.
- Failure to sign informed consent.
- Use of probiotics in the previous two weeks.

---

4. STUDY PROCEDURES

4.1 Intervention

Parents of patients meeting the inclusion criteria will be asked to enroll their child/ward in the study. Upon arrival at the emergency department (ED), patients will undergo a medical evaluation to determine whether antibiotic therapy is required. Patients will then be randomly assigned to either the intervention group or the placebo group. The intervention group will be asked to collect a stool sample within 24 hours of ED admission (T0) and, after collection, to begin a 14-day

regimen of the *Ofmom Synteract Bimbi* probiotic mixture (one sachet or five drops per day). At the end of the intervention period (T1), a second stool sample will be collected. The placebo group will also be required to provide two stool samples (T0 and T1) and will receive a placebo formulation consisting of Medium Chain Triglycerides (MCT) (one sachet or five drops per day). After 12 months, parents of participants who completed the 14-day regimen (either probiotic or placebo), provided both stool samples (T0 and T1), and submitted them for analysis will be contacted for a follow-up study. They will be asked to collect a third stool sample (T2) at the 12-month mark. The sample collection kit will either be provided at the time of recruitment in the ED or delivered to the participant's home. Parents will collect the stool sample at home, and they may either deliver the refrigerated sample to the ED during a scheduled follow-up visit or arrange for courier shipment of the sample.

4.2 Randomization

Enrolled subjects will be progressively assigned to a treatment group based on a computer-generated numerical sequence, with a 1:1 allocation ratio.

- 48 subjects will receive supplementation with *Ofmom Synteract Bimbi* (one sachet per day or five drops per day) (technical data sheet attached).
- 48 subjects will receive the placebo (technical data sheet attached).

Neither the investigator, the patient, the data manager, nor the personnel responsible for transportation will be aware of the treatment assignment. Both the probiotics and the placebo will be packaged in identical, neutral containers labeled only with the letters "A" or "B." The random allocation sequence will be generated by AAT, which will be responsible for safeguarding the randomization codes and conducting the decoding process at the end of the study.

4.3 Blinding

Participants, all healthcare professionals providing care, researchers, and data managers will remain blinded to the group assignment (placebo or intervention) throughout the duration of the trial.

5. ENDPOINTS

5.1 Primary Endpoint

Duration and degree of fever.

5.2 Secondary Endpoints

- Gut microbiota characterization through stool analysis using next-generation sequencing (metagenomics) and quantitative real-time PCR (qPCR) to identify variations in the major bacterial groups composing the microbiota at T0, T1, and, if applicable, T2. At T0, Lactobacilli and Bifidobacteria will also be isolated to further characterize the microbiota.
- Incidence of diarrhea in subgroup 1, consisting of patients undergoing antibiotic therapy, if prescribed by the emergency department physician.
- Number of patients in subgroup 2 who were not prescribed antibiotic therapy at T0 in the emergency department but received a prescription at a later time.

6. STUDY DURATION / TIMELINE

The study will have a total duration of 36 months, including 18 months for the enrollment phase and 6 months for the subsequent data analysis and report writing phase. The final 12 months will be necessary for organizing the follow-up with the third fecal sample collection, laboratory analysis, data analysis, and report writing. The expected duration of participation for each subject is 14 days. Refer to the attached CRF for the activities planned for each patient at the time of the ED visit. Fecal samples collected during the study will be sent to the AAT laboratory in agreed-upon batches between the Policlinico and the AAT facility.

7. STATISTICAL ANALYSIS

ISTITUTO DI RICOVERO E CURA A CARATTERE SCIENTIFICO DI NATURA PUBBLICA
Via Francesco Sforza, 28 - 20122 Milano
Tel. 02 5503.1
www.policlinico.mi.it
C.F. e P.I. 04724150968
Polo di ricerca, cura
e formazione universitaria
UNIVERSITÀ DEGLI STUDI DI MILANO

7.1 Sample Size The sample size calculation is based on the primary endpoint, specifically the duration of fever. The estimates are based on an average fever duration in children with upper respiratory tract infection in ED departments of 4 days (SD 2.6) (10). Data will be analyzed using intention-to-treat analysis. Assuming a reduction in fever duration in the intervention group by 37% (SD 1.5) and considering a Type I error of 0.05 and Type II error of 0.20, the calculated number of children to recruit is at least 48 per group.

7.2 Data Analysis Categorical or ordinal variables will be expressed as frequency (percentage, %); continuous variables as mean and standard deviation if normally distributed, and as median and interquartile range if not. Within-group and between-group comparisons will be performed using parametric or non-parametric statistical tests, as appropriate: for between-group comparisons, the Student's t-test (2 groups) or ANOVA (>2 groups) will be used for normally distributed continuous variables, the Mann-Whitney test (2 groups) or Kruskal-Wallis test (>2 groups) for asymmetrically distributed continuous variables, and the Chi-square test or Fisher's exact test (as appropriate) for categorical variables. The level of statistical significance is set at a p-value < 0.05, and where appropriate, 95% CIs will be calculated. Statistical software used will be SPSS (Statistical Package for Social Science version 20) and R Project for Statistical Computing.

Interim analyses will be performed to decide whether it is appropriate to terminate early or continue recruitment beyond the defined end of the trial. If there are deviations from the original statistical plan, the interim analyses will be described and justified in the final report, in the most appropriate manner.

8. ADVERSE EVENTS

The project does not involve the administration of drugs or other substances nor invasive clinical practices. Therefore, no adverse events are expected. Notification of any adverse events will follow current regulations in phytovigilance for products authorized for market release.

9. RISK/BENEFIT ASSESSMENT

The probiotic bacterial strains contained in the *Ofmom Synteract Bimbi* product (Lactobacillus rhamnosus HN0019, Bifidobacterium lactis HN001, and Bifidobacterium breve M16V) have been widely used for years in the Italian market as probiotic supplements by various companies. No risks have emerged related to the use of these strains in humans. The product's contribution to the patient is based on rebalancing their intestinal microbiota through a significant intake of bifidobacteria, which are a primary component of a child's intestinal microbiota, providing essential support for the digestion of breast milk and formula milk and for the proper development of the immune system. Therefore, it is believed that the intake of the probiotic mixture can help the child's immune system, both mucosal and systemic, to react to bacterial and viral infections.

10. STUDY MANAGEMENT

10.1 Data Collection and Management All data will be collected by designated evaluators using specifically developed data collection forms (Case Report Form, CRF). All collected data will be processed, analyzed, and stored confidentially. Each participant will be assigned a unique code at the time of enrollment. The file associating the participant's code with their identifying data will be stored separately on a password-protected computer accessible only to study personnel designated by the principal investigator. The data will be de-identified so that individuals accessing the database cannot trace the identity of the subjects. Only local investigators will be able to trace the identity of enrolled subjects.

10.2 Regulatory Aspects and Ethical Considerations

10.2.1 Competent Authority Approval In accordance with current regulations, the principal investigator must obtain approval from the appropriate Competent Authority before starting the clinical study. This study will be conducted in accordance with ICH/GCP (International Conference of Harmonization/Good Clinical Practice) guidelines and all applicable laws, including the Declaration of Helsinki of June 1964, as amended by the most recent World Medical Association General Assembly in Seoul, 2008.

10.2.2 Ethics Committee Approval The investigator must ensure that the protocol has been reviewed and approved by the local independent Ethics Committee (EC) before starting the study. The EC must also review and approve the informed consent form (IC) and all written information provided to the patient before enrollment in the study. If it is necessary to amend the protocol and/or IC during the study, the investigator will ensure the review and approval of such modified documents as requested by the EC. The content of such amendments will be implemented only after EC approval. Until then, the previously approved document version must be referred to.

10.2.3 Informed Consent (IC) The investigator or designated personnel is responsible for informing individuals about all aspects and procedures of the study. The investigator will seek the willingness to participate or not in the study and obtain informed consent. The process of obtaining informed consent must comply with current regulatory procedures. The investigator (or a designated collaborator) and the subject must date and sign the informed consent form before the patient initiates any study-related procedures. The subject will receive a copy of the IC dated and signed by both parties; the original copy will be stored in the study's designated archives. Neither the investigator nor designated personnel should in any way exercise any coercion or influence on a subject to induce them to participate or continue to participate in the study. A subject's decision to participate in the study must be completely voluntary. The investigator and designated personnel must emphasize to the subject that they can withdraw their consent at any time without any penalty or loss of any benefits they may be entitled to. Written or oral information about the study, including the written consent form, must not contain any language that coerces the subject to waive (even apparently) their legal rights or that exonerates the investigator, entity, or sponsor from liability for negligence. Patients involved in the study for a total period of 14 days will be asked to provide three fecal samples (T0, T1, and T2).

10.3 Investigator Responsibilities In accordance with applicable local regulations, the investigator must submit periodic reports on the progress of the study at their center to the EC and notify them of the study's closure. Periodic reports and closure notification are part of the investigator's responsibilities.

10.4 Study Monitoring In accordance with applicable regulations and good clinical practice (GCP), the monitor must visit or contact the center periodically. The duration, nature, and frequency of such visits/contacts depend on recruitment frequency, the quality of documents held by the center, and their adherence to the protocol.

Through such contacts, the monitor must:

- Check and evaluate study progress
- Review collected data
- Conduct source document verification
- Identify any problems and related solutions

The purposes of monitoring activities are to verify that:

- The rights and well-being of the subject are respected
- Study data are accurate, complete, and verifiable from original documents
- The study is conducted in accordance with the protocol and any approved amendments, GCP, and applicable regulations

The investigator must:

- Give the monitor direct access to all pertinent documentation
- Dedicate part of their time and staff to the monitor to discuss monitoring results and any other aspects.

The monitor must also contact the center before the study starts to discuss the protocol and data collection procedures with the staff.

10.5 Study Quality Assurance

As the sponsor, Fondazione IRCCS Ca' Granda, Ospedale Maggiore Policlinico may, at its discretion, conduct a quality control of the study. In this case, the investigator must allow the

monitor direct access to all pertinent documentation and dedicate part of their time and staff to the reviewer to discuss monitoring results and any other aspects of the study. Additionally, regulatory authorities may conduct inspections. In this case, the investigator must authorize the inspector direct access to all pertinent documentation and dedicate part of their time and staff to the inspector to discuss monitoring results and any other aspects of the study.

10.6 Study Closure Upon study closure, the monitor and investigator must activate a series of procedures:

- Review all study documentation
- Reconcile study data
- Reconcile all clarification reports

10.7 Document Archiving In accordance with current national regulations, the investigator must retain a copy of all documentation and store it in a dry and safe place after the study's closure.

10.8 Disclosure of Information Regarding Scientific Findings

10.8.1 Confidentiality The investigator and other personnel involved in the study must treat all study-related information (including the protocol, obtained data, and all documentation produced during the study) confidentially and must not use such information, data, or reports for purposes other than those described in the protocol. These restrictions do not apply to:

1. Information that becomes publicly available, not due to negligence by the investigator or their staff;
2. Information requiring confidential disclosure to the EC solely for the purpose of evaluating the study;
3. Information that must be disclosed to obtain appropriate medical care for a study subject.

10.8.2 Study Publications

Any publication of the study results (such as a manuscript or oral presentation) generated by the investigator must be submitted for review to the sponsor at least 60 days before submission for publication. The sponsor may require deletions and/or modifications if the publication contains confidential information or patentable material, which must be removed before publication. If the sponsor does not notify the investigator within the above 60 days of its objections, the publication can be submitted. If the sponsor provides comments within this period, the investigator must consider these comments in good faith. However, the investigator retains the right to submit the publication without the sponsor's consent. The study results will be published regardless of the study outcome.

REFERENCES

1. **Leclercq A, et al. (2016)**, Clinical Characteristics and Treatment Outcomes of Listeria monocytogenes Meningitis in France: The MONALISA National Prospective Cohort Study. The Lancet Infectious Diseases, 16(8), 887-897.
2. **Ricciotti, E., & Fitzgerald, G. A. (2011)**, Prostaglandins and Inflammation. Arteriosclerosis, Thrombosis, and Vascular Biology, 31(5), 986-1000.
3. **Bergogne-Bérézin, E. (1999)**, The Increasing Role of Anaerobes in Nosocomial Infections. Clinical Infectious Diseases, 28(1), 170-175.
4. **Fowler, V. G., et al. (2005)**, The Clinical and Microbiological Features of Staphylococcus aureus Bacteremia in a Prospective Study of 4,779 Episodes. Clinical Infectious Diseases, 41(5), 604-612.
5. **Weinstein, M. P., et al. (1997)**, The Clinical Significance of Positive Blood Cultures: A Comprehensive Analysis of 500 Episodes of Bacteremia and Fungemia in Adults. I. Laboratory and Epidemiologic Observations. Reviews of Infectious Diseases, 13(6), 792-802.
6. **Freeman, J., et al. (2010)**, The Changing Epidemiology of Clostridium difficile Infections. Clinical Microbiology Reviews, 23(3), 529-549.

7. **DeLaet, D. E., et al. (2006)**, Clinical and Economic Impact of Methicillin-Resistant Staphylococcus aureus Infections in New York City Hospitals. Emerging Infectious Diseases, 12(5), 692-698.
8. **Khanna, S., et al. (2012)**, Increased Incidence of Clostridium difficile Infection in Inflammatory Bowel Disease and Its Implications. Inflammatory Bowel Diseases, 18(11), 1998-2006.
9. **Vincent, J. L., et al. (2009)**, International Study of the Prevalence and Outcomes of Infection in Intensive Care Units. JAMA, 302(21), 2323-2329.
10. **Ruohola, A., et al. (2009)**, Acute Otitis Media and Respiratory Virus Co-Infection in Young Children. Pediatrics, 123(5), e639-e645.

APPENDIX

List of Abbreviations

- EC: Ethics Committee
- IC: Informed Consent
- CRF: Case Report Form
- GCP: Good Clinical Practice
- ED: Emergency Department
- PCR: Polymerase Chain Reaction
- SD: Standard Deviation

This document provides a comprehensive overview of the study design, objectives, procedures, and management strategies for the PROBIOS2021 study, ensuring a rigorous and ethically sound approach to evaluating the effects of probiotic supplementation in febrile children with upper respiratory tract infections.

ISTITUTO DI RICOVERO E CURA A CARATTERE SCIENTIFICO DI NATURA PUBBLICA
Via Francesco Sforza, 28 - 20122 Milano
Tel. 02 5503.1
www.policlinico.mi.it
C.F. e P.I. 04724150968
Polo di ricerca, cura e formazione universitaria
UNIVERSITÀ DEGLI STUDI DI MILANO

"Effetto della supplementazione di una miscela di ceppi probiotici (Bifidobacterium breve M-16V, Bifidobacterium lactis HN019, Lactobacillus rhamnosus HN001) in bambini febbrili con infezioni delle alte vie respiratorie"

Acronimo: PROBIOS2021

Promotore: Fondazione IRCCS Ca' Granda Ospedale Maggiore Policlinico,
Via Sforza 28, 20122 Milano, Italia

Centro coordinatore: PRONTO SOCCORSO PEDIATRICO
Fondazione IRCCS Ca' Granda Ospedale Maggiore Policlinico,
Via della Commenda 9, 20122 Milano, Italia

Sperimentatore Principale: Carlo Agostoni

Tipo di documento: protocollo di studio interventistico non farmacologico

Numero della versione: v.1.0

Data: 12/05/2021

DICHIARAZIONE DI RISERVATEZZA
Tutte le informazioni presentate in questo documento saranno considerate riservate e resteranno di proprietà esclusiva di Fondazione IRCCS Ca' Granda Ospedale Maggiore Policlinico. L'uso di tali informazioni riservate deve essere limitato al destinatario per lo scopo concordato e non deve essere divulgato, pubblicato o altrimenti comunicato a persone non autorizzate, per qualsiasi motivo, sotto qualsiasi forma senza il preventivo consenso scritto di Fondazione IRCCS Ca' Granda Ospedale Maggiore Policlinico.

FLOWCHART

	Assegnazione / randomizzazione		
Periodo	visita 1 (t0)	Fine dell'intervento (t1)	Follow up (t2)
Arruolamento			
consenso informato	x		x
criteri di inclusione/esclusione	x		
Compilazione CRF	x	x	
Somministrazione dell'intervento			
Gruppo con miscela di probiotici	x		
Gruppo placebo	x		
Rilevazioni			
Rilevazione variabili anamnestiche e antropometriche	x	x	
Misurazione della febbre	x	x	
Raccolta delle feci	x	x	x
Analisi microbiota intestinale	x	x	x

ISTITUTO DI RICOVERO E CURA A CARATTERE SCIENTIFICO DI NATURA PUBBLICA
Via Francesco Sforza, 28 - 20122 Milano
Tel. 02 5503.1
www.policlinico.mi.it
C.F. e P.I. 04724150968

Polo di ricerca, cura
e formazione universitaria
UNIVERSITÀ DEGLI STUDI DI MILANO

INDICE

1. INTRODUZIONE
- 1.1 Background e razionale

2. OBIETTIVO DELLA SPERIMENTAZIONE
- 2.1 Obiettivo primario
- 2.2 Obiettivo/i secondario/i

3. DISEGNO DELLO STUDIO
- 3.1 Disegno dello studio
- 3.2 Criteri d'inclusione
- 3.3 Criteri di esclusione

4. PROCEDURE RELATIVE ALLO STUDIO
- 4.1 Intervento
- 4.2 Randomizzazione
- 4.3 Cecità

5. ENDPOINT
- 5.1 Endpoint primario
- 5.2 Endpoint secondario

6. DURATA/TIMELINE DELLO STUDIO

7. ANALISI STATISTICA
- 7.1 Dimensione del campione
- 7.2 Analisi dei dati

8. EVENTI AVVERSI

9. VALUTAZIONE RISCHIO / BENEFICIO

10. GESTIONE DELLO STUDIO
- 10.1 Raccolta e gestione dei dati
- 10.2 Aspetti regolatori e considerazioni etiche
  - 10.2.1 Approvazione dell'Autorità competente
  - 10.2.2 Approvazione del Comitato Etico
  - 10.2.3 Consenso informato

ISTITUTO DI RICOVERO E CURA A CARATTERE SCIENTIFICO DI NATURA PUBBLICA
Via Francesco Sforza, 28 - 20122 Milano
Tel. 02 5503.1
www.policlinico.mi.it
C.F. e P.I. 04724150968

Polo di ricerca, cura e formazione universitaria
UNIVERSITÀ DEGLI STUDI DI MILANO

10.3 Doveri dello sperimentatore

10.4 Monitoraggio dello studio

10.5 Assicurazione della qualità dello studio

10.6 Chiusura dello studio

10.7 Archiviazione di documenti

10.8 Divulgazione di informazioni riguardanti la scoperta scientifica

10.8.1 Confidenzialità

10.8.2 Pubblicazioni

10.9 Diritti di proprietà intellettuale sui risultati dello studio

11. Indennità e risarcimento in caso di danni

12 Emendamenti al protocollo

13 Accordi finanziari

14 Diclosure sui conflitti di interesse

15 Referenze

16 Appendix

ISTITUTO DI RICOVERO E CURA A CARATTERE SCIENTIFICO DI NATURA PUBBLICA
Via Francesco Sforza, 28 - 20122 Milano
Tel. 02 5503.1
www.policlinico.mi.it
C.F. e P.I. 04724150968

Polo di ricerca, cura e formazione universitaria
UNIVERSITÀ DEGLI STUDI DI MILANO

Lista delle abbreviazioni

CE: Comitato Etico

CI: Consenso Informato

CRF: Case Report Form, scheda raccolta dati

GCP: Good Clinical Practice, buona pratica clinica

PS: Pronto Soccorso Pediatrico

PCR: Polymerase Chain Reaction

DS: Deviazione standard

Responsabilità (ruolo del promotore e dei collaboratori)

Carlo Agostoni: investigatore principale.

Collaborazioni esterne (analisi campioni biologici, analisi dati, procedure diagnostiche, etc.)

Istituzione	*Unità operativa*	*Nome partecipante*	*Ruolo e funzioni nello studio*
AAT – Advanced Analytical Technologies Srl, via P. Majavacca, 12 – 29017 Fiorenzuola d'Arda (Piacenza). Ph. 0523-461483.	*Research and Developement Lab*	*Dott. Lorenzo Morelli*<br>*Dott.ssa Marina Elli*	*Caratterizzazione del microbiota a partire dall'analisi fecale. Generazione della sequenza di allocazione random e decodifica finale*

Timelines

Stato dello studio	*Pianificazione*
Inizio dello studio	*Data 01/09/2021*
Chiusura dell'arruolamento	*Data 28/02/2023*
Report finale dello studio	*Data 31/08/2023*
Inizio follow-up a 12 mesi	*Data 01/05/2023*
Chiusura dell'arruolamento	*Data 31/05/2024*
Report finale dello studio	*Data 31/12/2024*

1. INTRODUZIONE

1.1 Background e razionale

L'organismo umano costituisce l'habitat di miliardi di microrganismi che nel loro insieme rappresentano un ecosistema chiamato *microbiota*. Il tratto gastrointestinale è il compartimento con più alta densità di cellule microbiche e, ad oggi, è la porzione più studiata (1). Il microbiota intestinale è sempre più considerato un "organo invisibile" del corpo umano, essenziale per la salute dell'ospite; tra batteri e ospite si viene a creare una simbiosi in cui l'organismo fornisce ai batteri i nutrienti necessari per la sua sopravvivenza e il suo sviluppo e i microrganismi producono sostanze necessarie per lo sviluppo e il benessere dell'individuo (2). Alla nascita, il tratto gastrointestinale del neonato è virtualmente sterile, ma viene subito esposto alla colonizzazione da parte di batteri opportunisti provenienti dal microbiota vaginale, fecale o associato alla pelle della madre; infatti, è stata riscontrata una significativa somiglianza tra la microflora del neonato e quella della madre, in linea con le precedenti evidenze di una trasmissione verticale dei microrganismi (3). La modalità di parto, quindi, condiziona la composizione della comunità microbiota del neonato. Durante le prime 24 ore di vita extrauterina, il tratto gastrointestinale viene colonizzato soprattutto da anaerobi facoltativi quali Streptococchi, Enterococchi ed Escherichia coli; successivamente, si insediano anche gli anaerobi obbligati come Bifidobatteri e Lactobacilli (4). Lo sviluppo della flora batterica è anche condizionato dall'alimentazione del neonato, quindi dal tipo di allattamento, al seno o attraverso formule artificiali. Numerosi studi hanno dimostrato la differente composizione del microbiota intestinale di soggetti allattati al seno rispetto a soggetti

allattati artificialmente. Il latte materno è ricco di monosaccaridi come lattosio, glicolipidi, glicoproteine, acido sialico, fucosio, oligosaccaridi e N-acetilglucosamina (3). Questi componenti, chiamati *prebiotici*, arrivano all'intestino non digeriti e svolgono una funzione di difesa creando uno scudo nei confronti di batteri patogeni. Inoltre, essendo i batteri in grado di attuare la fermentazione su composti del latte, si ha un aumento dei microrganismi fermentanti con una parallela diminuzione di quelli putrefattivi e una diminuzione del pH. In queste condizioni, si ha un miglioramento delle funzioni digestive e di assorbimento, con una relativa stimolazione del sistema immunitario e una riduzione del rischio di contrarre allergie e di carenze vitaminiche (5).

L'introduzione degli alimenti solidi, complementari al latte, è stata correlata alla riduzione della variabilità del microbiota intestinale tra i soggetti (6). Comunque, la maturazione della comunità microbica intestinale viene raggiunta, generalmente, intorno ai 2 anni di età (6,7). Il microbiota intestinale tende a mantenere un certo stato omeostatico nel corso della vita; quello del bambino a differenza di quello dell'adulto, che si ritiene essere relativamente stabile, è altamente dinamico e incline ad alterazioni. Numerosi sono i fattori che possono modulare tale maturazione, tra questi troviamo l'ambiente familiare in cui il bambino è inserito ed altre influenze legate alla cultura, alle tradizioni e all'area geografica di appartenenza.

Un altro fattore esterno che può determinare alterazione della composizione del microbiota è rappresentato dallo stato infiammatorio. L'infiammazione, infatti, è una risposta biologica all'attivazione del sistema immunitario dopo il contatto con agenti esterni infettivi.

I *probiotici* sono, secondo la definizione dell'OMS (Organizzazione Mondiale della Sanità) microrganismi vivi che, somministrati in quantità adeguata, apportano un beneficio alla salute dell'ospite (8). È noto come i *probiotici* siano in grado di rinforzare la risposta immunitaria, rispristinando la simbiosi all'interno del microbiota intestinale e coadiuvando, così, la protezione immunitaria nei confronti di diversi agenti patogeni, inclusi quelli responsabili delle infezioni respiratorie (9).

Infine, anche l'esposizione agli antibiotici, può alterare la composizione del microbiota intestinale. Gli antibiotici (sostanze prodotte da batteri, funghi, o di origine sintetica in grado di inibire la

crescita di altri microrganismi) sono i farmaci più comunemente utilizzati nelle popolazioni neonatale e pediatrica dei paesi occidentali. Terapie antibiotiche possono alterarne la composizione microbica di partenza e causare la perdita della resistenza alla colonizzazione (3). Di conseguenza, l'ospite può essere reso suscettibile alla colonizzazione da parte di uno o più agenti patogeni. Essendo la composizione del microbiota intestinale infantile molto importante per il mantenimento di un ottimo stato di salute, è altrettanto importante identificare accuratamente le specie batteriche residenti nel tratto gastrointestinale nei vari momenti di vita, sia in buono stato di salute che in condizioni patologiche. I primi anni di vita, quindi, risultano essere il periodo più critico e di fondamentale importanza per l'attecchimento del microbiota, che si accresce e si seleziona influenzando la formazione del sistema gastrointestinale, lo sviluppo del sistema immunitario, il mantenimento della barriera mucosale e l'assorbimento dei nutrienti.

2. OBIETTIVO/I O IPOTESI DELLA SPERIMENTAZIONE

La valutazione degli effetti della formulazione con probiotici *Ofmom Synteract Bimbi* sullo stato di salute del bambino. L'ipotesi è che i bambini che assumeranno la miscela avranno una durata e un'intensità della febbre minore rispetto al gruppo che assumerà il placebo. Inoltre, la formulazione probiotica potrà aiutare a prevenire i disturbi intestinali comunemente associati alla terapia antibiotica.

2.1 Obiettivo primario

Lo scopo primario dello studio è valutare gli effetti della formulazione con probiotici Ofmom Synteract Bimbi sull'intensità e la durata della febbre in risposta a infezioni delle vie aeree superiori.

2.2 Obiettivi secondari

Gli obiettivi secondari dello studio sono:

- Analisi dell'impatto dei prodotti probiotici sul microbiota intestinale dei soggetti trattati

- Analisi di differenti *outcomes* in sottogruppi di pazienti.

- Analisi del microbiota intestinale dopo 12 mesi dall'arruolamento.

3. DISEGNO DELLO STUDIO

3.1 Disegno dello studio

Studio interventistico prospettico biologico randomizzato controllato, non farmacologico

Rapporto di allocazione 1:1, in triplo cieco

La durata dello studio per ciascun paziente sarà di 14 giorni (somministrazione di *Ofmom Synteract Bimbi*, 1 stick al giorno, oppure 5 gocce al giorno). I probiotici verranno somministrati dopo la prima raccolta di un campione di feci. Lo studio verrà proposto ai genitori durante l'accesso al PS, ai quali verrà fatto compilare il consenso informato. Dopo 12 mesi, i genitori dei pazienti reclutati verranno ricontatatti e verrà proposto loro di eseguire la terza raccolta fecale (T2). Ai genirori verrà fatto compilare il consenso informato.

3.2 Criteri di inclusione

Saranno inclusi pazienti che soddisfano contemporaneamente i seguenti criteri e a cui verrà chiesto di partecipare in maniera volontaria:

- Accesso al PS con febbre e infezione delle vie aeree superiori
- Età > 28 giorni e ≤ 4 anni
- Firma del consenso informato da parte dei genitori, o del tutore

3.3 Criteri di esclusione

Saranno esclusi pazienti che possiedono almeno uno dei seguenti criteri:

- Presenza di patologie associate a immunosoppressione oppure trattamenti con immunosoppressori in corso
- Ospedalizzazione/ricovero
- Diarrea al momento dell'arruolamento
- Mancata firma del consenso informato

ISTITUTO DI RICOVERO E CURA A CARATTERE SCIENTIFICO DI NATURA PUBBLICA
Via Francesco Sforza, 28 - 20122 Milano
Tel. 02 5503.1
www.policlinico.mi.it
C.F. e P.I. 04724150968
Polo di ricerca, cura e formazione universitaria
UNIVERSITÀ DEGLI STUDI DI MILANO

- Uso di fermenti lattici/probiotici nelle precedenti 2 settimane

4. PROCEDURE RELATIVE ALLO STUDIO

4.1 Intervento

Ai genitori dei pazienti che presenteranno i criteri di inclusione verrà chiesto di far partecipare allo studio il proprio figlio/tutelato. Ai pazienti, giunti in PS, prima di avviare le procedure relative allo studio, su valutazione medica verrà prescritta o meno la terapia antibiotica. I pazienti verranno dunque allocati nel gruppo di intervento o nel gruppo placebo. Al gruppo di intervento sarà chiesto di raccogliere le feci entro 24 ore dall'accesso in PS (T0) e, dopo la raccolta, iniziare ad assumere per 14 giorni la miscela di probiotici *Ofmom Synteract Bimbi* (una bustina al giorno o 5 gocce al giorno). Alla fine dell'intervento (T1) sarà chiesta una nuova raccolta delle feci. Al gruppo placebo saranno richieste le due raccolte fecali (T0 e T1), e verrà somministrato il placebo, composto da MCT (*Medium Chain Triglycerides*) (una bustina al giorno o 5 gocce al giorno).

Dopo 12 mesi, i genitori dei soggetti che hanno assunto gocce o bustine per 14 giorni, raccolto e inviato entrambi i campioni fecali (T0 e T1), verranno ricontattaati e proposto loro di partecipare al follow up dello studio. Verrà chiesto di raccoligere un terzo campione fecale (T2) a distanza di 12 mesi. Il kit per la raccolta e spezizione dei campioni verrà fornito in occasione del reclutamento in PS oppure recapitato a domicilio. La raccolta fecale verrà eseguita dal genitore al proprio domicilio e il genitore stesso potrà portare il campione opportunatamente refrigerato al PS in occasione, se pianificata, di una visita di controllo, oppure verrà organizzata una spedizione, tramite corriere, del campione.

4.2 Randomizzazione

I soggetti arruolati verranno progressivamente assegnati al tipo di trattamento secondo una serie numerica generata da computer, rapporto di allocazione 1:1.

- 48 soggetti saranno supplementati con *Ofmom Synteract Bimbi* (una bustina al giorno, o 5 gocce al giorno) (allegata scheda tecnica).

- 48 soggetti assumeranno il placebo (allegata scheda tecnica).

Né lo sperimentatore, né il paziente, né il *data manager*, né chi si occuperà del trasporto sapranno l'assegnazione dell'intervento. Sia i probiotici che il placebo saranno contenuti in imballi identici e neutri, denominati solamente dalle lettere "A" o "B". La sequenza di allocazione random sarà generata dall'AAT, la quale si impegnerà a conservare i codici di randomizzazione e sarà il responsabile delle operazioni di decodifica al termine dello studio.

4.3 Cecità

I partecipanti, tutti i professionisti che erogano l'assistenza, i ricercatori e i *data manager* saranno mantenuti "ciechi" rispetto all'assegnazione del gruppo (placebo o intervento) durante tutta la durata del trial.

5.1 Endpoint primario: Durata e intensità della febbre

5.2 Endpoint secondari:

- Caratterizzazione del microbiota intestinale, attraverso analisi delle feci, attraverso *next generation sequencing* (*metagenomics*) and *real-time* PCR quantitativa, per identificare variazioni nei principali gruppi batterici che compongono il microbiota, tra T0, T1 ed eventuale T2. Al T0 verranno anche isolati lattobacilli e bifidobatteri, per meglio caratterizzare il microbiota.
- Incidenza di diarrea nel sottogruppo 1 di pazienti sottoposti a terapia antibiotica, se prescritta dal medico in PS.
- Numero di pazienti del sottogruppo 2 a cui non è stata prescritta terapia antibiotica in PS al T0 e ai quali verrà prescritta successivamente.

6. DURATA / TIMELINE DELLO STUDIO

Lo studio avrà la durata totale di 36 mesi, di cui 18 per la fase di arruolamento e 6 per la successiva fase di analisi dati e scrittura del report. Gli ultimi 12 mesi saranno necessari per l'organizzazione del follow up con terza raccolta fecale, analisi di laboratorio, analisi dei dati e scrittura del report. Durata prevista della partecipazione del singolo soggetto: 14 giorni. *Vedere CRF allegato per le attività previste per ciascun paziente al momento della visita in PS.* I campioni fecali raccolti nel

corso dello studio verranno inviati al laboratorio AAT nel corso dello studio stesso, in gruppi concordati tra il Policlinico e la struttura AAT.

7. ANALISI STATISTICA

7.1 Dimensione del campione

La dimensione del campione è stata calcolata sull'*endpoint* primario, in particolare sulla durata della febbre. Le stime sono basate su una durata media della febbre nei bambini con infezione delle alte vie respiratorie nei reparti di PS, di 4 giorni (2.6 DS) (10). I dati saranno analizzati mediante analisi *intention to treat*. Ipotizzando una riduzione della durata della febbre nel gruppo di intervento del 37% (1.5 DS) e considerando un errore di tipo I di 0.05 e di tipo II 0.20, il numero calcolato di bambini da reclutare è almeno di 48 per gruppo.

7.2 Analisi dei dati

Le variabili categoriche o ordinali saranno espresse come frequenza (percentuale, %); le variabili continue come media e deviazione standard, se normalmente distribuite, e come mediana e range interquartile se non. I confronti entro gruppo e tra gruppi saranno eseguiti con test di statistica parametrica o non parametrica, ove appropriato: per confronti tra gruppi, saranno utilizzati il test t di Student (2 gruppi) o l'ANOVA (>2 gruppi) per variabili continue distribuite normalmente, il test di Mann-Whitney (2 gruppi) o di Kruskal-Wallis (>2 gruppi) per variabili continue distribuite in modo asimmetrico, il test Chi quadrato o il test esatto di Fisher (ove appropriato) per le variabili categoriche. Il livello di significatività statistica è fissato ad un p-value < 0,05 e, dove appropriato, saranno calcolati gli IC95%. I software statistici utilizzati saranno SPSS (Statistical Package for Social Science versione 20) e R Project for Statistical Computing.

Saranno effettuate analisi intermedie (interim) per decidere se è opportuno interrompere precocemente o continuare il reclutamento oltre la fine definita del trial. Se dovessero esserci deviazioni dal piano statistico originale le analisi intermedie saranno descritte e giustificate nel rapporto finale, nella maniera più appropriata.

8. EVENTI AVVERSI

Il progetto non prevede somministrazione di farmaci o altre sostanze né pratiche cliniche invasive. Non sono quindi previsti eventi avversi. La notifica di eventuali eventi avversi seguirà la normativa vigente in ambito di fitosorveglianza per prodotti con autorizzazione all'immissione in commercio.

9. VALUTAZIONE RISCHIO / BENEFICIO

I ceppi batterici probiotici che sono contenuti nel prodotto Ofmom Synteract Bimbi (Lactobacillus rhamnosus HN0019, Bifidobacterium lactis HN001 e Bifidobacterium breve M16V) sono largamente impiegati da anni sul mercato italiano come integratori probiotici da parte di diverse aziende. Non sono emersi rischi legati all' impiego per l'uomo di questi ceppi. Il contributo che il prodotto potrà fornire al paziente si basa sul riequilibirio del suo microbiota intestinale mediante un significativo apporto di bifidobatteri che costituiscono una componente prioritaria del microbiota intestinale del bambino, che fornisce un supporto fondamentale per la digestione del latte materno e del latte in formula e per il corretto sviluppo del sistema immunitario. Per tale ragione si ritiene che l'assunzione della miscela probiotica possa aiutare il sistema immunitario del bambino, a livello mucosale e sistemico, a reagire alle infezioni batteriche e virali.

10. GESTIONE DELLO STUDIO

10.1 Raccolta e gestione dati

Tutti i dati saranno raccolti dai valutatori designati utilizzando moduli di raccolta dati elaborati appositamente (Case Report Form, CRF). Tutti i dati raccolti saranno trattati, analizzati e archiviati in maniera confidenziale. Ad ogni partecipante, al momento dell'arruolamento, verrà assegnato un codice univoco. Il file che associa il codice del partecipante con i relativi dati identificativi sarà conservato separatamente su un computer protetto da password ed accessibile al solo personale di studio designato dallo sperimentatore principale. La deidentificazione dei dati avverrà in maniera tale che le persone che accedono al database non potranno risalire in alcun modo all'identità dei soggetti. Solo gli sperimentatori locali potranno risalire all'identità dei soggetti arruolati.

ISTITUTO DI RICOVERO E CURA A CARATTERE SCIENTIFICO DI NATURA PUBBLICA
Via Francesco Sforza, 28 - 20122 Milano
Tel. 02 5503.1
www.policlinico.mi.it
C.F. e P.I. 04724150968
Polo di ricerca, cura e formazione universitaria
UNIVERSITÀ DEGLI STUDI DI MILANO

10.2 Aspetti regolatori e considerazioni etiche

10.2.1 Approvazione dell'Autorità Competente

In conformità con le normative vigenti, l'investigatore principale deve ottenere l'approvazione dall'appropriata Autorità Competente prima di iniziare lo studio clinico.

Questo studio sarà condotto in conformità con le regole dell'ICH / GCP (International Conference of Harmonization/Good Clinical Practice) e tutte le leggi applicabili, inclusa la Dichiarazione di Helsinki del giugno 1964, modificata dall'ultima World Medical Association General Assembly a Seoul, 2008.

10.2.2 Approvazione del Comitato Etico

Lo sperimentatore deve garantire che il protocollo sia stato visto e approvato dal Comitato Etico indipendente locale (CE) prima di iniziare lo studio.

Il CE deve anche verificare e approvare il modulo di consenso informato (CI) e tutte le informazioni scritte ricevute dal paziente prima dell'arruolamento nello studio.

Qualora fosse necessario modificare il protocollo e / o il CI durante lo studio, lo sperimentatore sarà il garante e quindi la persona incaricata di garantire la revisione e l'approvazione di tale documento modificato secondo richiesta del CE. Il contenuto di tali modifiche sarà implementato solo dopo che il CE le avrà approvate. Fino a quel momento, sarà necessario fare riferimento alla versione precedente del documento già approvato.

10.2.3 Consenso informato (CI)

Lo sperimentatore o altro personale da lui incaricato ha il compito di informare le persone su tutti gli aspetti e le procedure dello studio. Lo sperimentatore chiederà la volontà di partecipare o meno allo studio e raccoglierà il consenso informato.

Il processo per ottenere il consenso informato deve essere conforme alle procedure normative in vigore. L'investigatore (o un collaboratore designato) e il soggetto devono datare e firmare il modulo di consenso informato prima che il paziente avvii qualsiasi procedura relativa allo studio. Il soggetto riceverà una copia del CI datata e firmata da entrambe le parti; la copia originale sarà conservata negli archivi designati per lo studio. Né l'investigatore né il personale designato devono in alcun modo esercitare alcuna coercizione o influenza su un soggetto per

indurlo a partecipare o continuare a partecipare allo studio. La decisione di un soggetto di partecipare allo studio deve essere completamente volontaria. L'investigatore e il personale designato devono sottolineare al soggetto che può revocare il proprio consenso in qualsiasi momento senza alcuna penalità o perdita di qualsiasi beneficio a cui possa avere diritto.
Le informazioni scritte o orali relative allo studio, compreso il modulo di consenso scritto, non devono contenere alcuna espressione linguistica che costringa il soggetto a rinunciare (anche solo apparentemente) ai suoi diritti legali, o che esonererebbe lo sperimentatore, l'ente o lo sponsor da responsabilità per negligenza. Ai pazienti che saranno coinvolti nello studio per un periodo totale di 14 giorni, verranno chieste tre raccolte fecali (T0, T1 e T2).

10.3 Doveri dello sperimentatore

In conformità con le normative locali applicabili, lo sperimentatore deve inviare rapporti periodici riguardanti lo stato di avanzamento dello studio nel suo centro al CE e notificare allo stesso la chiusura dello studio. Le relazioni periodiche e la notifica di chiusura fanno parte delle responsabilità dello sperimentatore.

10.4 Monitoraggio dello studio

In conformità alle normative applicabili e la buona pratica clinica (GCP), il monitor deve visitare o contattare periodicamente il centro. La durata, la natura e la frequenza di tali visite / contatti dipendono dalla frequenza di reclutamento, dalla qualità dei documenti in possesso del centro e dalla loro adesione al protocollo.

Attraverso tali contatti, il monitor deve:

- controllare e valutare l'avanzamento dello studio
- esaminare i dati raccolti
- condurre la verifica del documento fonte
- identificare ogni problema e relative soluzioni

Gli scopi dell'attività di monitoraggio sono di verificare che:

ISTITUTO DI RICOVERO E CURA A CARATTERE SCIENTIFICO DI NATURA PUBBLICA
Via Francesco Sforza, 28 - 20122 Milano
Tel. 02 5503.1
www.policlinico.mi.it
C.F. e P.I. 04724150968
Polo di ricerca, cura e formazione universitaria
UNIVERSITÀ DEGLI STUDI DI MILANO

• i diritti e il benessere del soggetto siano rispettati
• i dati dello studio siano accurati, completi e verificabili dai documenti originali
• lo studio sia condotto in conformità al protocollo ed eventuali emendamenti approvati, GCP e le normative applicabili

Lo sperimentatore deve:
• dare al monitor l'accesso diretto a tutta la documentazione pertinente
• dedicare parte del suo tempo e del suo staff al monitor per discutere i risultati del monitoraggio e ogni altro possibile aspetto.
Il monitor deve inoltre contattare il centro prima dell'inizio dello studio per discutere il protocollo e le procedure di raccolta dei dati con lo staff.

10.5 Assicurazione della qualità dello studio

In qualità di promotore, Fondazione IRCCS Ca' Granda, Ospedale Maggiore Policlinico può effettuare a propria discrezione un controllo sulla qualità dello studio. In questo caso, lo sperimentatore deve consentire al monitor di accedere direttamente a tutta la documentazione pertinente e dedicare parte del suo tempo e del suo personale al revisore per discutere i risultati del monitoraggio ed eventuali altri aspetti dello studio.
Inoltre, le Autorità Regolatorie possono eseguire ispezioni. In questo caso, lo sperimentatore deve autorizzare all'ispettore l'accesso diretto a tutta la documentazione pertinente, e dedicare parte del suo tempo e del suo personale all'ispettore stesso per discutere i risultati del monitoraggio ed eventuali altri aspetti dello studio.

10.6 Chiusura dello studio

Al momento della chiusura dello studio, il monitor e lo sperimentatore devono attivare una serie di procedure:
• rivedere tutta la documentazione dello studio
• riconciliare i dati dello studio
• conciliare tutte le relazioni di chiarimento.

ISTITUTO DI RICOVERO E CURA A CARATTERE SCIENTIFICO DI NATURA PUBBLICA
Via Francesco Sforza, 28 - 20122 Milano
Tel. 02 5503.1
www.policlinico.mi.it
C.F. e P.I. 04724150968
Polo di ricerca, cura e formazione universitaria
UNIVERSITÀ DEGLI STUDI DI MILANO

10.7 Archiviazione di documenti

In conformità con le vigenti normative nazionali, lo sperimentatore deve conservare una copia di tutta la documentazione e conservarla in un luogo asciutto e sicuro dopo la chiusura dello studio.

10.8 Divulgazione di informazioni riguardanti la scoperta scientifica

10.8.1 Confidenzialità

Lo sperimentatore e altro personale coinvolto nello studio deve trattare tutte le informazioni relative allo studio (compreso il protocollo, i dati ottenuti e tutta la documentazione prodotta nel corso dello studio) e non deve utilizzare tali informazioni, dati o relazioni per scopi diversi da quelli descritti nel protocollo. Queste restrizioni non si applicano a:

1) informazioni che diventano pubblicamente disponibili, non a causa di negligenza da parte dello sperimentatore o del suo personale;

2) informazioni che richiedono la divulgazione riservata a CE al solo scopo di valutare lo studio;

3) informazioni che devono essere divulgate al fine di ottenere cure mediche adeguate per un soggetto di studio.

10.8.2 Pubblicazioni

La Fondazione IRCCS Ca' Granda, Ospedale Maggiore Policlinico è l'unico proprietario dei dati. Lo sperimentatore, le persone della Fondazione da lui incaricate e i ricercatori dell'AAT – Advanced Analytical Technologies Srl, potranno accedere al dataset finale del trial. Il responsabile scientifico della sperimentazione si impegna alla stesura del rapporto finale e a renderne pubblici i risultati. I risultati saranno pubblicati in modo anonimo e presentati in modalità aggregata.

10.9 Diritti di proprietà intellettuale sui risultati dello studio

Il Promotore dello Studio e AAT – Advanced Analytical Technologies Srl, che collabora allo Studio in qualità di laboratorio centralizzato presso i cui spazi verranno analizzati campioni biologici raccolti, si danno reciprocamente atto sin da ora che nel corso dello Studio, nei limiti di

quanto strettamente necessario allo svolgimento di questo, potrebbero essere utilizzati in varia misura, informazioni, dati, know-how, materiale tecnico e/o beni protetti da diritti di proprietà industriale e/o intellettuale o suscettibili di protezione, sviluppati prima dell'avvio dello Studio da ciascuna parte ("Background"). Le parti si riconoscono pertanto un diritto reciproco per l'utilizzo del proprio Background, laddove necessario, per la sola conduzione dello Studio e nei limiti della sua durata. Resta inteso che la suddetta licenza d'uso del Background non comporta l'attribuzione di alcun diritto ulteriore sul Background che resta quindi nella titolarità esclusiva della parte concedente.

Tutti i dati e i risultati scaturiti dalle attività di ricerca condotte durante lo Studio saranno di proprietà esclusiva di Fondazione, Promotore dello Studio.

11. INDENNITÀ E RISARCIMENTI IN CASO DI DANNI

In caso di eventi indesiderati o eventuali danni che dovessero derivare dalla partecipazione alla ricerca, la Polizza Assicurativa del nostro Istituto è estesa anche alla copertura dei soggetti partecipanti ai progetti di ricerca.

12. EMENDAMENTI AL PROTOCOLLO

///

13. ACCORDI FINANZIARI

L'azienda Ofmom Srl fornirà in maniera gratuita il prodotto Ofmom Synteract Bimbi, in due formulazioni: stick e gocce. L'azienda Coree srl sosterrà le spese di analisi, di invio dei materiali e finanzierà eventuali costi per predisporre personale addetto alla ricerca, attraverso una donazione liberale di denaro alla Fondazione IRCCS Ca' Granda Ospedale Maggiore Policlinico.

14. DISCLOSURE SUI CONFLITTI DI INTERESSE

I ricercatori dichiarano l'assenza di conflitti di interesse.

15. REFERENCES

1. Wilks, M. (2007). Bacteria and early human development. Early human development, 83(3), 165-170.
2. Jandhyala, S. M., Talukdar, R., Subramanyam, C., Vuyyuru, H., Sasikala, M., & Reddy, D. N. (2015). Role of the normal gut microbiota. World journal of gastroenterology: WJG, 21(29), 8787.
3. Palmer, C., Bik, E. M., DiGiulio, D. B., Relman, D. A., & Brown, P. O. (2007). Development of the human infant intestinal microbiota. PLoS biol, 5(7), e177.
4. Duncan, S. H., Hold, G. L., Harmsen, H. J., Stewart, C. S., & Flint, H. J. (2002). Growth requirements and fermentation products of Fusobacterium prausnitzii, and a proposal to reclassify it as Faecalibacterium prausnitzii gen. nov., comb. nov. International journal of systematic and evolutionary microbiology, 52(6), 2141-2146.
5. Collins, M. D., & Gibson, G. R. (1999). Probiotics, prebiotics, and synbiotics: approaches for modulating the microbial ecology of the gut. The American journal of clinical nutrition, 69(5), 1052s-1057s.
6. Roger, L. C., & McCartney, A. L. (2010). Longitudinal investigation of the faecal microbiota of healthy full-term infants using fluorescence in situ hybridization and denaturing gradient gel electrophoresis. Microbiology, 156(11), 3317-3328.
7. Magne, F., Hachelaf, W., Suau, A., Boudraa, G., Mangin, I., Touhami, M., ... & Pochart, P. (2006). A longitudinal study of infant faecal microbiota during weaning. FEMS microbiology ecology, 58(3), 563-571
8. FAO/WHO working group. (2001). Report of a joint FAO/WHO expert consultation on evaluation of health and nutritional properties of probiotics in food including powder milk with live lactic acid bacteria. Córdoba, Argentina (October 1–4, 2001).
9. Shahbazi, R., Yasavoli-Sharahi, H., Alsadi, N., Ismail, N., & Matar, C. (2020). Probiotics in Treatment of Viral Respiratory Infections and Neuroinflammatory Disorders. Molecules, 25(21), 4891.
10. 10. Thompson, M., Vodicka, T. A., Blair, P. S., Buckley, D. I., Heneghan, C., & Hay, A. D. (2013). Duration of symptoms of respiratory tract infections in children: systematic review. Bmj, 347, f7027).
