# Supplementary material for: Probiotics and Fever Duration in Children With Upper Respiratory Tract Infections: A Randomized Clinical Trial
Source: JAMA Netw Open. 2025 Mar 14;8(3):e250669. doi: 10.1001/jamanetworkopen.2025.0669 (PMC11909606; doi:10.1001/jamanetworkopen.2025.0669)
Supplement: Supplement 2. — eTable 1. Baseline Characteristics of Children Included in the Total Sample, Intention-to-Treat (ITT) Analysis and Per-Protocol (PP) Analysis eTable 2. Demographic Characteristics of Dropout and Compliant Subjects eTable 3. Per Protocol Analysis (Median Difference) eTable 4. Differences Between Fever Duration Between Placebo and Probiotic Groups Considering as Outcome the Number of Days With Fever After Enrollment eTable 5. Poisson Regression Model of Probiotics on Duration of Fever After Enrollment [file jamanetwopen-e250669-s002.pdf]

Supplemental Online Content

Bettocchi S, Comotti A, Elli M, et al. Probiotics and fever duration in children with upper respiratory tract infections: a randomized clinical trial. *JAMA Netw Open*. Published online March 14, 2025. doi:10.1001/jamanetworkopen.2025.0669

eTable 1. Baseline Characteristics of Children Included in the Total Sample, Intention-to-Treat (ITT) Analysis and Per-Protocol (PP) Analysis

eTable 2. Demographic Characteristics of Dropout and Compliant Subjects

eTable 3. Per Protocol Analysis (Median Difference)

eTable 4. Differences Between Fever Duration Between Placebo and Probiotic Groups Considering as Outcome the Number of Days With Fever After Enrollment

eTable 5. Poisson Regression Model of Probiotics on Duration of Fever After Enrollment

This supplemental material has been provided by the authors to give readers additional information about their work.

© 2025 Bettocchi S et al. *JAMA Network Open*

eTable 1. Baseline Characteristics of Children Included in the Total Sample, Intention-to-Treat (ITT) Analysis and Per-Protocol (PP) Analysis

	**Total sample N=128**		**ITT sample N=87**		**PP sample N=70**	
	Placebo group (n=65)	Probiotic group (n=63)	Placebo group (n=50)	Probiotic group (n=37)	Placebo group (n=43)	Probiotic group (n=27)
Age, mean (SD), y	2.5 (1.3)	2.6 (1.3)	2.5 (1.3)	2.5 (1.4)	2.5 (1.3)	2.5 (1.4)
Sex						
Male	36 (55)	33 (52)	29 (58)	19 (51)	26 (60)	13 (48)
Female	29 (45)	30 (48)	21 (42)	18 (49)	17 (40)	14 (52)
Race and ethnicity[a]						
Asian	1 (1.5)	4 (6)	1 (2)	2 (5)	1 (2)	2 (7.5)
Eurasian	1 (1.5)	1 (2)	1 (2)	1 (3)	1 (2)	1 (4)
Caucasian	52 (80)	48 (76)	42 (84)	30 (81)	36 (84)	22 (81)
Hispanic	7 (11)	6 (10)	3 (6)	3 (8)	2 (5)	2 (7.5)
North-African	4 (6)	4 (6)	3 (6)	1 (3)	3 (7)	NA
Nutritional status[b]						
Normal weight	60 (92)	58 (92)	45 (90)	32 (87)	38 (88)	23 (85)
Underweight	3 (5)	1 (2)	3 (6)	1 (3)	3 (7)	1 (4)
Overweight	2 (3)	4 (6)	2 (4)	4 (10)	2 (5)	3 (11)
N days with fever before enrollment, median (IQR)	2 (1–3)	2 (1–3)	2 (1–3)	2 (1–3)	2 (1–3)	2 (1–3)
Antibiotic at enrollment	16 (25)	17 (27)	16 (32)	15 (41)	14 (33)	11 (41)

Abbreviation: NA, not applicable

Data are N (%), unless otherwise indicated.

[a]Race and ethnicity were self-identified by parents or caregivers

[b]The World Health Organization (WHO) reference charts were used to calculate Z-scores and 3 percentiles for weight for age, weight for length, and body mass index (calculated as weight in 4 kilograms divided by height in meters squared). WHO criteria were consulted to classify child nutritional status.

eTable 2. Demographic Characteristics of Dropout and Compliant Subjects

	**Dropout (n=41)**	**Compliant (n=87)**	***P* value[a]**
Age, mean (SD), y	2.5 (1.3)	2.5 (1.3)	.94
Sex			
Male	21 (50)	48 (55)	.82
Female	20 (50)	39 (45)	NA
Race and ethnicity			
Asian	2 (5)	3 (3)	.19
Eurasian	NA	2 (2)	NA
Caucasian	28 (68)	72 (83)	NA
Hispanic	7 (17)	6 (7)	NA
North-African	4 (10)	4 (5)	NA

Abbreviation: NA, not applicable,

Data are N (%), unless otherwise indicated. Parents reported their children’s ethnicity.

[a] *P* value was calculated using chi-square test in case of percentage differences and t-test in case of mean difference.

Per-protocol analysis

We performed per protocol analysis including only fully compliant subjects.

© 2025 Bettocchi S et al. *JAMA Network Open*

eTable 3. Per Protocol Analysis (Median Difference)

Group	Total No. (%)	Fever duration, median (IQR)	*P* value[a]
Total sample	70 (100)	4 (3–5)	NA
Placebo group	43 (61)	5 (4–6)	<.001
Probiotic group	27 (39)	3 (2–4)	NA

Abbreviation: NA, not applicable.
[a]Probiotic vs placebo groups. *P* value was calculated using Wilcoxon rank-sum
RR (95% CI)=0.62 (0.48, 0.79)

<u>Primary outcome as fever after enrollment</u>

eTable 4. Differences Between Fever Duration Between Placebo and Probiotic Groups Considering as Outcome the Number of Days With Fever After Enrollment

	Total No. (%)		Fever duration after enrollment, median (IQR)		*P* value[a]
Group	PP	ITT	PP	ITT	
Total sample	70 (100)	87 (100)	2 (1–4)	2 (1–3)	NA
Placebo group	43 (61)	50 (57)	3 (2–4.5)	2.5 (1–4)	.001
Probiotic group	27 (39)	37 (43)	1 (1–2)	1 (1–2)	NA

Abbreviation: NA, not applicable.
[a]Probiotic vs placebo groups. *P* value was calculated using Wilcoxon rank-sum tests

eTable 5. Poisson Regression Model of Probiotics on Duration of Fever After Enrollment

	RR (95% CI)
Per Protocol	0.43 (0.30, 0.61)
Intention-To-Treat	0.41 (0.29, 0.57)

RR are adjusted by sex, age, antibiotic intake, and number of days with fever before enrollment.

© 2025 Bettocchi S et al. *JAMA Network Open*
