# Supplementary material for: Probiotics and Fever Duration in Children With Upper Respiratory Tract Infections: A Randomized Clinical Trial
Source: JAMA Netw Open. 2025 Mar 14;8(3):e250669. doi: 10.1001/jamanetworkopen.2025.0669 (PMC11909606; doi:10.1001/jamanetworkopen.2025.0669)
Supplement: Supplement 3. — Data Sharing Statement [file jamanetwopen-e250669-s003.pdf]

Data Sharing Statement

Bettocchi. Probiotics and Fever Duration in Children With Upper Respiratory Tract Infections. *JAMA Netw Open*. Published March 14, 2025. doi:10.1001/jamanetworkopen.2025.0669

Data
Additional Information: ClinicalTrials.gov, https://clinicaltrials.gov/study/NCT06052540?cond=NCT06052540&rank=1, Identifier: NCT06052540
Data available: Yes
Data types: Deidentified participant data
How to access data: https://redcap.policlinico.mi.it
When available: With publication

Supporting Documents
Document types: None

Additional Information
Who can access the data: researchers whose proposed use of the data has been approved
Types of analyses: for a specified purpose
Mechanisms of data availability: with a signed data access agreement
